# The AI risk repository: A meta-review, database, and taxonomy of risks from artificial intelligence

**DOI:** 10.1016/j.patter.2026.101517

**Published:** 2026-03-30

**Authors:** Peter Slattery, Alexander K. Saeri, Emily A.C. Grundy, Jess Graham, Michael Noetel, Risto Uuk, James Dao, Soroush Pour, Stephen Casper, Neil Thompson

**Affiliations:** 1 MIT FutureTech, Massachusetts Institute of Technology, Cambridge, MA 02139, USA; 2 School of Psychology, The University of Queensland, St. Lucia, QLD 4072, Australia; 3 Future of Life Institute, 1040 Brussels, Belgium; 4 KU Leuven, 3000 Leuven, Belgium; 5 Harmony Intelligence, Sydney, NSW, Australia; 6 Computer Science and Artificial Intelligence Laboratory, Massachusetts Institute of Technology, Cambridge, MA 02139, USA

**Keywords:** AI governance, societal impact of AI, AI safety, risk assessment, machine-learning governance

## Abstract

The risks posed by artificial intelligence (AI) concern academics, auditors, policymakers, AI companies, and the public. Researchers, policymakers, and technology companies discuss AI risks using inconsistent terminology—the same word may describe different problems, while different words describe identical concerns. This fragmentation impedes coordinated responses to AI challenges. We address this by creating the AI Risk Repository: a living database of 1,725 risks extracted from 74 existing taxonomies and frameworks. We organize these risks using two complementary classification systems. The Causal Taxonomy classifies risks by their origins: which entity causes them (human or AI), whether intentional, and when they occur (before or after deployment). The Domain Taxonomy classifies risks by their effects across seven areas, from discrimination and privacy violations to misinformation and weapons development. This shared reference enables more coordinated approaches to discussing, researching, auditing, and governing AI systems across sectors and jurisdictions.

## Highlights

• A comprehensive AI Risk Database: 1,725 risks from 74 frameworks
• Existing taxonomies split risk attribution between humans (38%) and AI models (42%)
• In those taxonomies, 13% of categorized risks relate to the pre-deployment phase
• We proposed a domain taxonomy mapping AI risks across seven societal impact areas

## The bigger picture

Artificial intelligence (AI) is reshaping society, from video generation to medical diagnosis, coding agents to autonomous vehicles. Yet researchers, policymakers, and technology companies lack shared terminology for discussing AI risks. Consider “privacy”: one framework uses this term to describe a model’s ability to leak sensitive training data, while another uses it to mean freedom from government surveillance. Conversely, researchers have introduced “Goodhart’s law,” “specification gaming,” “reward hacking,” and “mesa-optimization” to describe the same phenomenon of AI systems optimizing for measured proxies rather than intended goals. This terminological diversity creates friction: comparing findings across studies requires mapping between frameworks, and comprehensive risk coverage requires consulting multiple taxonomies that use different organizing principles. This paper addresses this challenge by creating a comprehensive catalog of AI risks. We systematically analyzed every major AI risk framework published to date—74 frameworks containing 1,725 distinct risks—and organized them into a unified system. Our two classification systems reveal important patterns: contrary to common assumptions, human decisions cause nearly as many AI risks (38%) as the AI systems themselves (42%). The work provides practical tools for anyone working on AI safety, from developers conducting risk assessments to policymakers writing regulations to auditors evaluating AI systems. By establishing a common reference point, this repository creates the foundation for more coordinated and comprehensive approaches to managing AI’s risks while realizing its benefits.

## Teaser

Slattery et al. systematically analyzed 74 AI risk frameworks containing 1,725 distinct risks to create the AI Risk Repository. They developed two complementary taxonomies—a Causal Taxonomy (classifying risks by entity, intent, and timing) and a Domain Taxonomy (organizing risks across seven societal impact areas)—providing a unified foundation for AI risk assessment, governance, and auditing.

## Introduction

For humanity to reap the benefits of artificial intelligence (AI), it must understand and address its risks. The range of possible risks is wide, from plagiarism^1^ to pandemics.^2^ Understanding and categorizing AI risks is fundamental to technological forecasting and anticipating the societal trajectory of AI. As AI systems become increasingly autonomous and capable, the ability to forecast their potential impacts requires a comprehensive understanding of the risk landscape. Naturally, these risks have drawn considerable attention from academics, regulators, policymakers, and the public.^3^^,^^4^^,^^5^^,^^6^ However, that broad attention has led to a diverse and disparate set of taxonomies, classifications, and other lists of AI risks. This paper aims to collate all of these taxonomies and harmonize them into one living resource (http://airisk.mit.edu) to hold and organize those risks.

Having a range of partially overlapping risk frameworks can lead to confusion and hide vulnerabilities. For example, organizations developing AI models may present risk-mitigation plans that lack detail^7^ or address relatively few risks (cf. Anthropic, Google DeepMind, and OpenAI^8^^,^^9^^,^^10^). Similarly, risk evaluators may be less able to comprehensively evaluate and report on AI risks without a clear understanding of the full range of threats (cf. Nevo et al.^11^). A full taxonomy can help companies, governments, and model developers prioritize and know where to place controls.

Another challenge arising from multiple, overlapping frameworks is the conceptual ambiguity akin to psychology’s “jingle jangle fallacies,”^12^ whereby people use the same name for different risks or different names for the same risk. For example, “privacy” might refer to a model’s ability to leak sensitive information from the training data^13^ or being free from government surveillance,^14^ which are very different risks. Our repository addresses this by providing a richer clustering of risk terms, mapping how different frameworks carve up the risk landscape and enabling practitioners to identify which specific risks underlie broader categories. We aim to provide additional structure that supports cross-framework comparison and comprehensive coverage. Shared understanding can reduce confusion and promote research usage, cross-study comparison, and the development of cumulative knowledge (e.g., Marcolin et al.^15^ and Harrison McKnight and Chervany^16^). Coherent frameworks are also important in legal, political, and practical settings, where they are often cited as goals for regulatory processes.^17^ For example, the United States (US)-European Union (EU) Trade and Technology Council stated in its joint roadmap for trustworthy AI and risk management, “shared terminologies and taxonomies are essential for operationalizing trustworthy AI and risk management in an interoperable fashion.”^18^

Previous attempts to find shared understanding have usually drawn from narrative reviews rather than the result of a systematic search. The few systematic reviews that exist for exceptions^19^^,^^20^ have focused on specific categories of AI system (generative AI and artificial general intelligence [AGI], respectively) rather than risks from AI systems broadly. Existing taxonomies vary considerably in their adherence to best practice criteria for classification systems.^21^ We observed that many taxonomies prioritize comprehensiveness over mutual exclusivity (with risks spanning multiple categories), while others achieve parsimony at the cost of exhaustive coverage. Few taxonomies describe explicit revision processes and most are descriptive rather than explanatory in orientation. The number of competing taxonomies inadvertently makes it challenging to integrate relevant research into a cohesive shared understanding.

In this paper, we aim to address these limitations. We systematically reviewed existing AI risk classifications, frameworks, and taxonomies. We extracted the categories and subcategories of risks from included reports into a living database that we have updated over time (http://airisk.mit.edu). We applied a “best-fit” framework synthesis approach^22^^,^^23^ to develop two complementary taxonomies. The Causal Taxonomy captures antecedent conditions for risk: which entity’s actions led to the risk, whether intentional, and when in the development life cycle it occurs. The Domain Taxonomy captures consequent harms: the domains of impact such as loss of control, weapons development, privacy, economic harm, and so on. Together, these taxonomies allow risks to be classified by both their origins and their effects. We sought to rigorously extract AI risk frameworks into a comprehensive, extensible, and categorized risk database. This creates a foundation for a more coherent and complete approach to managing the risks posed by AI systems.

This work makes several theoretical contributions to technological forecasting and social change. First, we provide a comprehensive empirical foundation for theories of emerging technology risk by systematically analyzing 74 frameworks encompassing 1,725 distinct risks. This moves beyond single-framework approaches to create a meta-theoretical structure for understanding technological risk.

Second, our dual-taxonomy approach—combining causal and domain-based classifications—advances theoretical understanding of how technological risks emerge and manifest in society. The Causal Taxonomy provides a descriptive framework for categorizing how existing taxonomies attribute risk sources. We understand this framework simplifies a more complex reality: many risks emerge from interactions between human decisions and AI system behaviors rather than from either in isolation.^24^ Our “Other” category (21% of coded risks) captures cases where this attribution was ambiguous or explicitly interactional, although existing taxonomies rarely theorized these interaction effects explicitly.

Third, we found that certain risk categories appear in relatively few frameworks (e.g., AI welfare appeared in 3% of frameworks and multi-agent risks in 7%). This suggests that future taxonomies might need to expand coverage as new risks like these are identified.

## Results

### Systematic literature search

**Figure 1:**
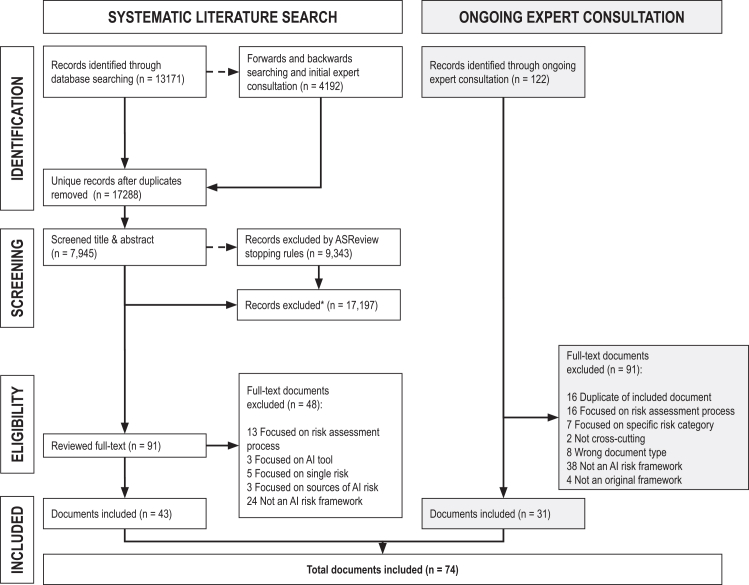
PRISMA flow diagram for systematic literature search, ongoing expert consultation, and screening Flow diagram illustrating the identification, screening, eligibility assessment, and inclusion of documents for the AI Risk Repository. The left pathway shows the systematic literature search, which identified 17,288 unique records from database searching (*n* = 13,171) and forward/backward searching with initial expert consultation (*n* = 4,192). Title and abstract screening using ASReview software reduced this to 91 full-text documents, of which 43 met inclusion criteria. The right pathway shows ongoing expert consultation (May 2024 to December 2025), which identified 122 additional documents through recommendations, with 31 meeting inclusion criteria after full-text review. Combined, 74 documents were included in the final analysis. Exclusion reasons at the full-text stage included focus on risk-assessment processes rather than risk classification, focus on single risk categories, wrong document type, and documents that were not original AI risk frameworks. ∗The ASReview software was used to assist in screening titles and abstracts from searches.

We retrieved 17,288 unique articles from our searches and expert consultations. Of these records, we screened 7,945. We excluded 9,343 via our stopping criteria while using ASReview, which used machine learning to determine when further screening was unlikely to yield relevant content. We assessed the full text of 91 articles. A total of 43 articles and reports met the eligibility criteria: 21 from our search, 13 from forward and backward searching, and 9 from expert suggestions (Figure 1). A database of included records and extracted data is available on the Open Science Framework.^25^

### Ongoing expert consultation

In the period May 2024 to March 2025, we received recommendations to consider 44 documents. Of those, 22 met eligibility criteria. Between March 2025 and December 2025, we received 78 document recommendations and included 9 that met criteria.

### Characteristics of included documents

We included 74 documents comprising 25 peer-reviewed articles, 26 preprints, 8 conference papers, and 15 reports. We mainly identified recent literature, with all but five (93%) of the included documents published later than 2020.

The included documents varied in the type or scope of AI they focused on. In most cases, the type of AI was not explicitly defined (*n* = 27). Large language model was the next most common (*n* = 13), followed by generative AI (*n* = 10), general-purpose AI (*n* = 8), AGI (*n* = 4), and machine learning (*n* = 3). Other terms included “AI and machine learning” (always described in the document as “AI/ML”), AI assistant, algorithmic systems, frontier AI, advanced AI, conversational agents, and embodied AI.

Together, the included documents presented a total of 1,725 risk categories (e.g., “privacy risks”) or subcategories of risk (e.g., “compromising privacy by leaking sensitive information”^26^). Not all documents presented eligible risk categories in sufficient detail to allow us to code them with our taxonomies; two included documents were not coded as having any distinct risk categories or framework,^27^^,^^28^ so were not extracted. Our supplemental information also includes a database of all risks and the included documents and frameworks (Tables S1 and S10).

### Causal Taxonomy of AI risks

We created our taxonomies using a best-fit framework synthesis. To create the Causal Taxonomy of AI risks (Table 1), we selected and iterated on a taxonomy of causal factors.^29^ This taxonomy uses Entity, Intent, and Timing to classify risks in the AI Risk Database. We coded 1,480 of the 1,725 (86%) potential risks extracted from our documents against the Causal Taxonomy. 136 did not present sufficient information to assess the Entity, Intent, or Timing, and 87 were discarded as they did not fit our definition of risk (e.g., where a previous taxonomy said “governance - regulation” without describing how the regulation itself was a risk^19^). Other reasons included the risk descriptions being too broad to code (e.g., “damage to political and economic institutions”). In the supplemental information, we provide detailed descriptions of each causal taxonomy variable (Note S1), report the iterative development of the best-fit taxonomy (Note S3), and present coverage of the included documents across the Causal Taxonomy (Tables S2, S3, and S4).

**Table 1:** Causal Taxonomy of AI risks

Category	Level	Description
Entity	human	the risk is caused by a decision or action made by humans
	AI	the risk is caused by a decision or action made by an AI system
	other	the risk arises from human-AI interaction rather than either agent alone, or the causing entity is ambiguous or unspecified
Intent	intentional	the risk occurs due to an expected outcome from pursuing a goal
	unintentional	the risk occurs due to an unexpected outcome from pursuing a goal
	other	the risk is presented as occurring without clearly specifying the intentionality
Timing	pre-deployment	the risk occurs before the AI is deployed
	post-deployment	the risk occurs after the AI model has been trained and deployed
	other	the risk occurs across both pre- and post-deployment phases or is presented without a clearly specified time of occurrence

The Causal Taxonomy classifies AI risks according to three categories of antecedent factors. Entity specifies whether the risk is caused by decisions or actions made by humans or by AI systems, or arises from human-AI interaction (or is ambiguously specified). Intent specifies whether the risk occurs as an expected outcome (intentional) or unexpected outcome (unintentional) of pursuing a goal or is presented without clear specification of intentionality. Timing specifies whether the risk occurs before deployment (pre-deployment), after the AI model has been trained and deployed (post-deployment), or spans both phases (or is unspecified). Each risk is classified under exactly one level within each category. Of 1,725 extracted risks, 1,480 (86%) contained sufficient information to be coded against this taxonomy. See also Table S1 for the full distribution of risks across taxonomy categories.

### Domain Taxonomy of AI risks

To create the Domain Taxonomy of AI risks, we used the best-fit framework synthesis to iterate on a taxonomy of categories of AI risk.^26^ This taxonomy catalogs hazards and harms associated with AI. We coded 1,506 (87%) of the 1,725 potential risks extracted from our documents against the Domain Taxonomy. As above, the rest did not contain the necessary details or did not fit our definition of “risk.”

In Table 2, we present the Domain Taxonomy including subdomains and short descriptions of each subdomain. In the supplemental information, we present a detailed description of each subdomain using information from included documents (Note S2) as well as analysis of the distribution of risks and domains across the database (Tables S5, S6, S7, S8, and S9). We also transparently report the evolution of the best-fit taxonomy.

**Table 2:** Domain Taxonomy of AI risks

Domain/subdomain		Description
**1**	**Discrimination and toxicity**	
1.1	Unfair discrimination and misrepresentation	unequal treatment of individuals or groups by AI, often based on race, gender, or other sensitive characteristics, resulting in unfair outcomes and unfair representation of those groups
1.2	Exposure to toxic content	AI that exposes users to harmful, abusive, unsafe. or inappropriate content. May involve providing advice or encouraging action. Examples of toxic content include hate speech, violence, extremism, illegal acts, or child sexual abuse material, as well as content that violates community norms such as profanity, inflammatory political speech, or pornography
1.3	Unequal performance across groups	accuracy and effectiveness of AI decisions and actions is dependent on group membership, where decisions in AI system design and biased training data lead to unequal outcomes, reduced benefits, increased effort, and alienation of users
**2**	**Privacy and security**	
2.1	Compromise of privacy by obtaining, leaking, or correctly inferring sensitive information	AI systems that memorize and leak sensitive personal data or infer private information about individuals without their consent. Unexpected or unauthorized sharing of data and information can compromise user expectation of privacy, assist identity theft, or cause loss of confidential intellectual property
2.2	AI system security vulnerabilities and attacks	vulnerabilities that can be exploited in AI systems, software development toolchains, and hardware, resulting in unauthorized access, data and privacy breaches, or system manipulation causing unsafe outputs or behavior
**3**	**Misinformation**	
3.1	False or misleading information	AI systems that inadvertently generate or spread incorrect or deceptive information, which can lead to inaccurate beliefs in users and undermine their autonomy. Humans that make decisions based on false beliefs can experience physical, emotional, or material harms
3.2	Pollution of information ecosystem and loss of consensus reality	highly personalized AI-generated misinformation that creates “filter bubbles” where individuals only see what matches their existing beliefs, undermining shared reality and weakening social cohesion and political processes
**4**	**Malicious actors and misuse**	
4.1	Disinformation, surveillance, and influence at scale	using AI systems to conduct large-scale disinformation campaigns, malicious surveillance, or targeted and sophisticated automated censorship and propaganda, with the aim of manipulating political processes, public opinion, and behavior
4.2	Cyberattacks, weapon development or use, and mass harm	using AI systems to develop cyber weapons (e.g., by coding cheaper, more effective malware), develop new or enhance existing weapons (e.g., lethal autonomous weapons or chemical, biological, radiological, nuclear, and high-yield explosives), or use weapons to cause mass harm
4.3	Fraud, scams, and targeted manipulation	using AI systems to gain a personal advantage over others such as through cheating, fraud, scams, blackmail, or targeted manipulation of beliefs or behavior. Examples include AI-facilitated plagiarism for research or education, impersonating a trusted or fake individual for illegitimate financial benefit, or creating humiliating or sexual imagery
**5**	**Human-computer interaction**	
5.1	Overreliance and unsafe use	anthropomorphizing, trusting, or relying on AI systems by users, leading to emotional or material dependence and to inappropriate relationships with, or expectations of, AI systems. Trust can be exploited by malicious actors (e.g., to harvest information or enable manipulation) or result in harm from inappropriate use of AI in critical situations (e.g., medical emergency). Over-reliance on AI systems can compromise autonomy and weaken social ties
5.2	Loss of human agency and autonomy	delegating by humans of key decisions to AI systems, or AI systems that make decisions that diminish human control and autonomy, potentially leading to humans feeling disempowered, losing the ability to shape a fulfilling life trajectory or becoming cognitively enfeebled
**6**	**Socioeconomic and environmental harm**	
6.1	Power centralization and unfair distribution of benefits	AI-driven concentration of power and resources within certain entities or groups, especially those with access to or ownership of powerful AI systems, leading to inequitable distribution of benefits and increased societal inequality
6.2	Increased inequality and decline in employment quality	social and economic inequalities caused by widespread use of AI, such as by automating jobs, reducing the quality of employment, or producing exploitative dependencies between workers and their employers
6.3	Economic and cultural devaluation of human effort	AI systems capable of creating economic or cultural value, including through reproduction of human innovation or creativity (e.g., art, music, writing, coding, and invention), destabilizing economic and social systems that rely on human effort. The ubiquity of AI-generated content may lead to reduced appreciation for human skills, disruption of creative and knowledge-based industries, and homogenization of cultural experiences
6.4	Competitive dynamics	competition by AI developers or state-like actors in an AI “race” by rapidly developing, deploying, and applying AI systems to maximize strategic or economic advantage, increasing the risk they release unsafe and error-prone systems
6.5	Governance failure	inadequate regulatory frameworks and oversight mechanisms that fail to keep pace with AI development, leading to ineffective governance and the inability to manage AI risks appropriately
6.6	Environmental harm	the development and operation of AI systems that cause environmental harm, such as through energy consumption of data centers or the materials and carbon footprints associated with AI hardware
**7**	**AI system safety, failures, and limitations**	
7.1	AI pursuing its own goals in conflict with human goals or values	AI systems that act in conflict with ethical standards or human goals or values, especially the goals of designers or users. These misaligned behaviors may be introduced by humans during design and development, such as through reward hacking and goal misgeneralization, and may result in AI using dangerous capabilities such as manipulation, deception, or situational awareness to seek power, self-proliferate, or achieve other goals
7.2	AI possessing dangerous capabilities	AI systems that develop, access, or are provided with capabilities that increase their potential to cause mass harm through deception, weapons development and acquisition, persuasion and manipulation, political strategy, cyber-offense, AI development, situational awareness, and self-proliferation. These capabilities may cause mass harm due to malicious human actors, misaligned AI systems, or failure in the AI system
7.3	Lack of capability or robustness	AI systems that fail to perform reliably or effectively under varying conditions, exposing them to errors and failures that can have significant consequences, especially in critical applications or areas that require moral reasoning
7.4	Lack of transparency or interpretability	challenges in understanding or explaining the decision-making processes of AI systems, which can lead to mistrust, difficulty in enforcing compliance standards or holding relevant actors accountable for harms, and the inability to identify and correct errors
7.5	AI welfare and rights	ethical considerations regarding the treatment of potentially sentient AI entities, including discussions around their potential rights and welfare, particularly as AI systems become more advanced and autonomous
7.6	Multi-agent risks	risks from multi-agent interactions due to incentives (which can lead to conflict or collusion) and/or the structure of multi-agent systems, which can create cascading failures, selection pressures, new security vulnerabilities, and a lack of shared information and trust

The Domain Taxonomy classifies AI risks into seven domains and 24 subdomains based on the types of hazards and harms they describe. Domain 1 (Discrimination and toxicity) includes unfair discrimination, exposure to toxic content, and unequal performance across groups. Domain 2 (Privacy and security) covers privacy compromise and AI system security vulnerabilities. Domain 3 (Misinformation) addresses false information and pollution of the information ecosystem. Domain 4 (Malicious actors and misuse) encompasses disinformation at scale, cyberattacks and weapons, and fraud and manipulation. Domain 5 (Human-computer interaction) includes over-reliance and loss of human agency. Domain 6 (Socioeconomic and environmental harm) covers power centralization, inequality, devaluation of human effort, competitive dynamics, governance failure, and environmental harm. Domain 7 (AI system safety, failures, and limitations) addresses misalignment, dangerous capabilities, lack of robustness, lack of transparency, AI welfare, and multi-agent risks. Unlike the Causal Taxonomy, domains are not mutually exclusive; some risks span multiple domains. Of 1,725 extracted risks, 1,506 (87%) were coded against this taxonomy. See also Table S2 for subdomain descriptions with examples from included documents.

### Overlap between new and existing taxonomies

The Causal Taxonomy demonstrates that current frameworks cover multiple causal factors—the extracted risks were nearly equally attributed to AI systems (42%) versus human decisions (38%), and similarly distributed between intentional (35%) and unintentional (35%) causes. Frameworks tended to focus on post-deployment risks (62%), with fewer addressing pre-deployment risks (13%) and 25% either spanning both phases or not specifying timing.

The Domain Taxonomy analysis reveals substantial variation in the comprehensiveness of existing frameworks. While certain domains (e.g., AI system safety) were represented in over 75% of the taxonomies we reviewed, categories such as AI welfare and rights (present in only 3% of frameworks) and multi-agent risks (7% of frameworks) were rarely incorporated. Frameworks typically addressed only a subset of risks—averaging 8 of our identified 24 subdomains, with coverage ranging from 1 to 20 subdomains. Such variability indicates that existing taxonomies generally provide partial rather than comprehensive risk categorizations, potentially leaving significant gaps in risk-assessment and management frameworks.

## Discussion

This paper presents attempts to rigorously curate AI risk frameworks into a comprehensive, extensible, and categorized risk database. We developed two taxonomies to classify 1,725 risks from 74 documents: the Causal Taxonomy of AI risks (addressing how, when, and why risks emerge) and the Domain Taxonomy of AI risks (categorizing risks into seven domains and 24 subdomains).

Our repository provides essential infrastructure for forecasting risks from emerging AI technologies that extend beyond current systems. The field is characterized by rapid growth—93% of included documents were published after 2020, with most appearing as pre-prints or conference papers. This suggests urgency in knowledge dissemination but poses challenges for coordination and standardization. Our extensible risk database aims to facilitate that coordination by continually arranging those risks into useful taxonomies.

As AI evolves toward greater autonomy, multi-modal capabilities, and agentic behaviors, new risk categories will emerge at the intersections of our identified domains. For instance, the convergence of advanced language models with robotic systems may create novel risks spanning our “AI system safety, failures and limitations” and “human-computer interaction” domains. Multi-agent risks appeared in only 7% of current frameworks. Given the trajectory toward AI systems that interact autonomously with each other and with human society,^30^ future frameworks may benefit from greater attention to this category.

The Causal Taxonomy reveals patterns in where prior frameworks anticipate risks. The distribution of risks in our database shows greater attention to post-deployment risks (62%) than to pre-deployment risks (13%). The appropriate distribution would depend on empirical evidence about risk frequency and severity that is beyond the scope of this review. Still, this distribution may warrant attention, because excessive focus on post-deployment risks may blind us to threats from development itself.^31^

The Domain Taxonomy reveals uneven coverage across risk categories. While “AI system safety, failures, and limitations” and “socioeconomic and environmental harm” are discussed in over 75% of documents, emerging areas like “AI welfare and rights” (3% of documents) and “multi-agent risks” (7% of documents) are not yet integrated into frameworks, despite their potential significance as AI systems become more autonomous and interconnected. We hope one function of our review is to reduce the duplication (e.g., of new taxonomies including “socioeconomic and environmental harm”) while aiming for comprehensive coverage (e.g., including emerging areas such as AI welfare).

While our repository catalogs risks, it simultaneously enables innovation by providing clear boundaries within which beneficial AI development can proceed. Understanding the full risk landscape allows developers to channel innovation toward addressing genuine human needs while avoiding harmful pathways. For instance, awareness of discrimination risks has spurred innovation in fairness-aware machine learning, while understanding of AI misalignment drives research into frontier model evaluations and alignment. Our comprehensive mapping reveals “safe harbors” for innovation—areas where risks are well understood and manageable—as well as frontier domains requiring careful exploration. By making risks explicit and categorical, we reduce uncertainty for innovators and investors, potentially accelerating beneficial AI deployment. The repository thus serves a dual function: protecting society from AI harms while enabling the realization of AI’s transformative potential for addressing societal challenges from healthcare to climate change.

### Practical implications for technology management

Our AI Risk Repository provides concrete tools for managing AI’s societal impact across multiple stakeholder groups.

• **For technology managers and AI developers**: the repository supports comprehensive risk assessment during AI development and deployment. Organizations can use our causal analysis to identify opportunities for mitigations, recognizing that many “AI risks” actually require human intervention during design, development, or governance phases. The Domain Taxonomy enables systematic risk assessment during product development cycles. Organizations can use our 24 subdomains as a checklist during design reviews, ensuring comprehensive risk consideration before deployment. We recommend integrating our taxonomies into existing risk-management frameworks, using them to structure safety cases and inform resource allocation decisions. For example, the Causal Taxonomy reveals that 13% of identified risks relate to the pre-deployment phase. Organizations concerned with development-phase safety may find this subset of the repository particularly relevant for structuring their risk assessments.
• **For policymakers and regulators**: the repository forms the basis for operationalizing vague regulatory references to “harm” and “risk.” It can support compliance frameworks required by regulations such as the EU AI Act^32^ and facilitate international collaboration through shared terminologies—essential for initiatives like the EU-US Trade and Technology Council’s efforts to develop interoperable AI governance.^18^ The repository operationalizes vague regulatory concepts such as “high-risk AI systems” by providing 1,725 specific risk examples organized into actionable categories. Regulators can use the Causal Taxonomy to design targeted interventions—for instance, focusing on the 37% of risks attributed to human decisions through training requirements while addressing the 42% from AI system actions through technical standards.
• **For auditors and compliance officers**: the repository addresses a critical gap: the absence of comprehensive frameworks for determining when and where AI systems pose specific risks. Current industry risk-management frameworks often address narrow risk subsets, making comprehensive evaluation challenging.^33^ Our repository provides the foundation for developing objective standards necessary for comprehensive AI audits. The Domain Taxonomy’s seven domains can structure audit protocols, while the repository’s specific risk examples can inform testing scenarios. Organizations can demonstrate due diligence by documenting how they address each relevant subdomain.
• **For researchers**: the taxonomies enable systematic synthesis across disparate studies and identification of knowledge gaps. The database can guide research prioritization—for instance, our finding that pre-deployment human-caused risks receive minimal attention despite emerging concerns about dangerous AI development. It may provide a framework for prioritizing among the full range of AI risks and help researchers identify the controls necessary to cover that range.

### Limitations and future directions

Several limitations warrant consideration. While we attempted to extract risks verbatim and conducted extensive calibration, conducting extraction and coding in duplicate would reduce the risk of bias or error. Our search, while comprehensive, focused on cross-cutting frameworks and excluded domain-specific taxonomies. Users focused on a particularly narrow domain (e.g., medical diagnostics) might benefit from using those focused taxonomies. We have not conducted a formal validation study to assess whether independent users can reliably categorize novel risks using our taxonomies. Future work should assess inter-rater reliability with coders external to the authorship team. Such validation would increase confidence in the taxonomies’ utility for standardizing risk discourse across organizations. Our Causal Taxonomy assigns risks to human or AI sources, which simplifies the sociotechnical reality that many risks emerge from human-AI interactions rather than from either agent in isolation.^24^ Similarly, our “Timing” categories (pre-deployment, post-deployment, and other) simplify a more complex reality. Modern AI development often involves continuous iteration: models are deployed, monitored, retrained, and updated in ongoing cycles rather than progressing through discrete phases. The repository’s structure also trades some precision for comprehensiveness; while we aimed to capture all risks, we could not capture risk likelihood, severity, or interactions between risks.

The field would benefit from assessing the severity and likelihood of these risks, as has been done for catastrophic AI risks.^34^ Doing so would help in prioritizing among these risks, prioritizing among proposed controls, and addressing underexplored areas. Future research will need to help actors understand which of these risks are most important for them to focus on. Once prioritized, it is still unclear how companies and governments should best mitigate those risks without stifling innovation. Future studies should aim to identify the best mitigations for the most important risks. Finally, any new frameworks should consider including a broader range of risks identified here. For example, most frameworks focus on language models rather than emerging concerns like agentic AI and multi-agent systems.^14^^,^^20^ The limited attention to AI welfare and rights (appearing in only two documents) deserves attention until we can confidently rule out AI sentience for increasingly advanced systems.^35^

### Conclusions

Our AI Risk Repository provides critical infrastructure for effective technology governance in an era of rapid AI advancement. The development of appropriate governance frameworks requires a shared understanding of which risks need to be governed, and our repository provides this foundation. The repository enables adaptive governance approaches essential for managing rapidly evolving technologies. Rather than static regulatory frameworks, our living database supports dynamic policymaking that can respond to emerging risks while avoiding premature or overly broad restrictions.

The taxonomies bridge multiple research domains essential for understanding AI’s societal impact. From a forecasting perspective, our systematic categorization of 1,725 risks provides the empirical foundation for projecting AI’s future trajectories. From a management perspective, our taxonomies offer practical tools for organizational decision-making about AI development and deployment. From an impact assessment perspective, our comprehensive risk mapping enables evaluation of AI’s multi-faceted effects across social, economic, and environmental dimensions. From a governance perspective, our repository provides the shared terminology and categorization schemes necessary for effective policy development. This integration across domains is essential because AI risks themselves transcend traditional boundaries—a single AI system may simultaneously raise concerns about discrimination (social impact), competitive dynamics (economic impact), and existential risks.

The AI Risk Repository establishes a foundation for more coordinated approaches to understanding and managing AI risks. By making the database living and extensible, we enable continuous refinement as new risks emerge and understanding evolves. It provides a common reference point that can reduce conceptual confusion, guide research and policy priorities, and support the development of comprehensive governance frameworks. As AI capabilities advance rapidly, such shared understanding becomes essential for managing the risks so that we can safely realize the benefits.

## Methods

**Figure 2:**
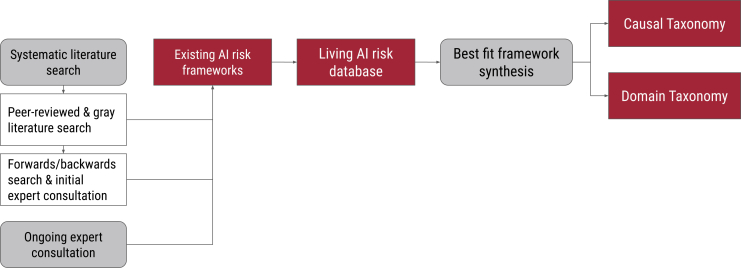
Overview of study methodology Schematic overview of the study methodology, showing the progression from systematic literature search to taxonomy development. The systematic literature search comprised three components: peer-reviewed and gray literature search, forward/backward search and initial expert consultation, and ongoing expert consultation. These searches identified existing AI risk frameworks, which were extracted into a living AI Risk Database. Best-fit framework synthesis was then applied to develop two complementary outputs: the Causal Taxonomy (classifying risks by entity, intent, and timing) and the Domain Taxonomy (classifying risks by type of harm across seven domains).

A summary of our methodology is available in Figure 2. We used a systematic search strategy, forward and backward searching, and expert consultation to identify AI risk classifications, frameworks, and taxonomies. Since conducting the original systematic literature search, we have periodically identified additional relevant research through an ongoing expert consultation. We extracted the individual risks from these documents into a living AI Risk Database (http://airisk.mit.edu). We conducted two best-fit framework syntheses to create a Causal Taxonomy (Table 1) and Domain Taxonomy (Table 2) of AI risks by adapting existing frameworks.^26^^,^^29^ We did this by testing their effectiveness at coding our risk data and modifying them until we created a final version that could effectively code all relevant risks.

### Systematic literature search

We conducted this study as a systematic review.^36^^,^^37^ The protocol was registered in advance using the Open Science Framework in April 2024 (https://osf.io/egzc8). We included reviews, articles, reports, and documents primarily focused on proposing new frameworks, taxonomies, or other structured classifications of risks from AI present across multiple locations and industry sectors. We followed the Society for Risk Analysis^38^ in defining “AI risk” as “the possibility of an unfortunate occurrence associated with the development or deployment of artificial intelligence,” while recognizing that this term can be defined in many ways.^39^^,^^40^

We excluded book chapters, theses, commentaries, editorials, and protocols. Our pilot searches suggested that including these documents would significantly increase the number of search results to screen but not the number of relevant results. We excluded documents that discussed impacts, outcomes, or other consequences of AI without specifying specific risks because we were interested in risk classification.

Due to our interest in broad, structured classifications of risks from AI, we excluded documents that focused only on risks from AI that are present in a single location or sector or that discussed risks specific to particular risk categories (e.g., content solely focused on different types of unfair decision-making) or very specific AI tools (e.g., content solely focused on risks from DALL-E). We excluded content that merely cited or discussed existing theories, frameworks, models, taxonomies, and other structured classifications rather than proposing and explaining them, because we wanted to understand and extract specific risks using their original source material. We excluded anything that discussed sources of risk at a high level of abstraction (e.g., the sources of sociotechnical risk in AI) or risk-assessment processes (e.g., how organizations can assess risks from AI) rather than focusing on classifying AI risks more specifically. Non-English articles, reports, and documents were excluded due to resource constraints related to their retrieval and translation.

Two of the above exclusion criteria were added after protocol registration in order to retain only the most relevant documents: (1) focus only on one category of risk from AI and (2) focus on sources of risk or the risk-assessment process.

### Search strategy

Our search strategy comprised two stages. In stage 1, we conducted a systematic search of peer-reviewed and gray literature (i.e., non peer-reviewed materials) to identify relevant articles. We begin by explaining our search-term generation and strategy, followed by our database searches in Scopus and various pre-print databases. We then describe our screening process, which uses active learning with ASReview. This process includes four phases: initial random screening for training data, application of active learning with specific stopping rules, model switching for comprehensive coverage, and quality evaluation. Finally, we outline our full-text screening and calibration procedures. In stage 2, we conducted forward (citation) and backward (references) searching and expert consultation to identify additional eligible articles (both processes described below).

### Stage 1: Searching and screening peer-reviewed and gray literature

#### Searching

Search terms were generated through an iterative process and chosen for their empirical balance between sensitivity and specificity.^41^ This included terms related to AI (Artificial intelligence, AI, Artificial general intelligence, AGI); frameworks, taxonomies, and other structured classifications (Framework, Review, Overview, Taxonomy∗); and risks (Risk, Harm, Hazard). This led to the following search string:

TITLE-ABS-KEY (( “artificial intelligence” OR ai OR “artificial general intelligence” OR agi) AND (framework OR taxonom∗ OR review) AND (risk OR harm OR hazard) ) AND (LIMIT-TO ( LANGUAGE, “English”) ).

We conducted a Scopus search to identify relevant academic research. The same search string was used on the following pre-print databases to identify relevant literature: arXiv, Social Science Research Network (SSRN), Research Square, medRxiv, TechRxiv, bioRxiv, and ChemRxiv. Both searches were conducted on April 4, 2024. Relevant articles were downloaded for screening.

#### Title/abstract and full-text screening

Two authors formed a team to conduct title/abstract and full-text screening. Before screening, the team calibrated their decision-making by independently screening the same randomly selected articles (*n* = 23), comparing the results, and resolving disagreements. Agreement was achieved on 21 of 23 records (91%). To expedite title and abstract screening, we used active learning in ASReview,^42^ with the process done independently and in duplicate by two reviewers.

Active learning is an emerging research technique that uses machine learning to reduce the total number of records requiring manual screening. It is now widely used for efficiently screening large datasets in systematic reviews and meta-analyses^43^^,^^44^ and has been validated in a number of diverse fields^42^^,^^44^ and datasets.^45^

Throughout the active-learning process, we followed the four-step SAFE procedure outlined by Boetje and van de Schoot^46^ to ensure that screening identified relevant articles both rigorously and efficiently.

##### Phase 1: Screen a random set of articles to create training data for active-learning model

As per SAFE, the screening team each randomly screened and labeled 1% of the total search yield (264 records in total). Each member of the team then created separate projects in ASReview and uploaded their own files, which included all retrieved studies and the random screening data. The random screening data were automatically marked as prior knowledge, and the active-learning phase commenced.

##### Phase 2: Apply active learning during screening until stopping rule is reached

For the first iteration of the active-learning model, the team followed the recommendation of Boetje and van de Schoot^46^ to use the Oracle model and the default model setup (TF-IDF as the feature extractor, Naive Bayes as the classifier, maximum as the query strategy, and dynamic resampling [double] as the balance strategy). We aimed to follow 4-fold stopping heuristics according to Boetje and van de Schoot,^46^ screening until four mutually independent conditions are met.

(1) All key papers are marked as relevant.
(2) At least twice the estimated number of relevant records in the total dataset are screened.
(3) More than 10% of the total dataset has been screened.
(4) No relevant records are identified in the last 50 records screened.

These four stopping heuristics aim to achieve a sensitivity of 95%,^44^ ensuring comprehensive data assessment while preventing excessive time spent on unlikely candidates.

The team met three of these conditions: they (1) screened more than twice the estimated number of relevant records, (2) screened more than 10% of the total dataset, and (3) had not identified any relevant records in the last 50 records. However, one condition (“all key papers are marked as relevant”) could not be met due to a bug with the model. Only three out of the four key papers^20^^,^^26^^,^^47^^,^^48^ had appeared in the screening process, and the final key paper was scheduled to appear several thousand papers later. Because stage 3 of the SAFE process aims to ensure that records are not missed due to the initial model, the screening team switched models to find out whether a new model would locate the relevant paper.

##### Phase 3: Switch active-learning model and screen additional records until stopping rule is reached

Based on a review of relevant literature (van de Schoot et al^42^; e.g., Campos et al.^44^), we use the Oracle model with the following setup: a fully connected neural network (two hidden layers) model as the classifier and sBert as the feature extractor, maximum as the query strategy, and dynamic resampling (double) as the balance strategy. The model was trained on the data that were labeled while using the previous model. Screening stopped when no extra relevant records were identified in the last 50 records. Both authors screened in the missing key paper were within the first two records found by the new model.

##### Phase 4: Evaluate quality

For quality checks, the screening team screened records previously labeled as irrelevant using the Oracle model and the default model setup (i.e., the same model that was used in the initial/main model phase). This model was trained using the ten highest- and lowest-ranked records from the model switching phase. Both team members screened records to identify any relevant records that might have been falsely excluded. This continued until the stopping rule was met (no extra relevant records identified in the last 50 records).

One member of the screening team screened the full text of all records that were included at the title/abstracts step. For calibration, 10% of the records were screened in duplicate, with 100% inter-rater reliability achieved. Conflicts were resolved by discussion for any remaining records.

### Stage 2: Forward and backward searching and expert consultation

Following full-text screening, we undertook forward and backward searching using Scopus, Google Scholar, and various pre-print servers hosting the included gray literature. Backward searching involved identifying and reviewing all references from articles included in stage 1, while forward searching involved identifying and reviewing all articles that cited an included article. We also undertook an expert consultation, which involved sharing the preliminary set of included articles with their authors and other experts and requesting recommendations for relevant frameworks that had been overlooked. All records identified during forward and backward searching and expert consultation were screened by one author. Those that met inclusion criteria were added to the backlog for extraction.

### Extraction into living AI Risk Database

Five authors were involved in data extraction. A template data-extraction spreadsheet was developed to capture various details from the studies, including title, abstract, author, year, source/outlet, risk category name, risk category description, risk subcategory name, risk subcategory description, and page number. This spreadsheet was refined over several rounds of pilot testing and extractor calibration on subsets of randomly selected articles. Data extraction was then conducted individually, with regular meetings for discussion and conflict resolution. Based on the recommendations of grounded theory, we aimed to capture the studied phenomena directly from the data rather than impose our interpretations (Corbin and Strauss,^49^ cf. Charmaz^50^). Consequently, we extracted risks based on how the authors presented them, maintaining fidelity to their original categorizations and descriptions.

### Best-fit framework synthesis approach

**Figure 3:**
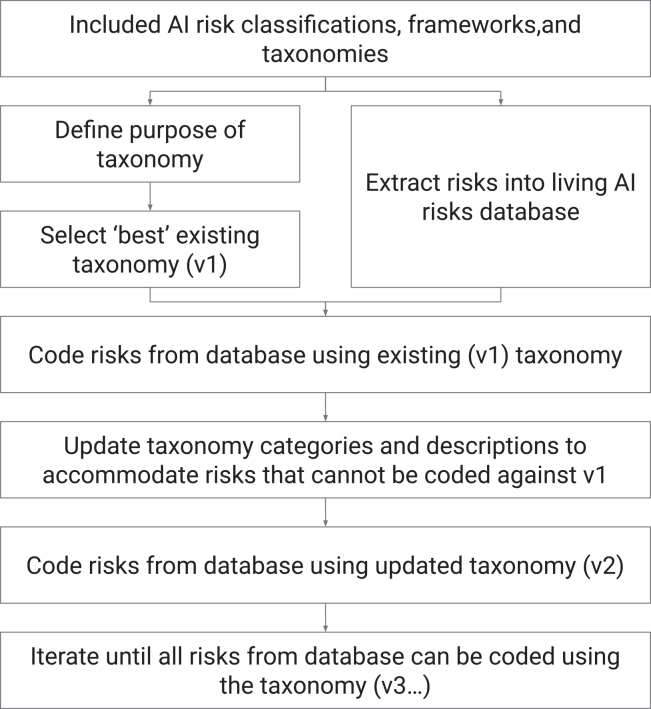
Methodology for best-fit framework synthesis Flowchart illustrating the iterative best-fit framework synthesis process used to develop the Causal and Domain taxonomies. The process began with included AI risk classifications, frameworks, and taxonomies. Two parallel initial steps involved defining the purpose of each taxonomy and extracting risks into the living AI risks database. An existing taxonomy was selected as the starting framework (v1). Risks from the database were coded using this existing taxonomy. Categories and descriptions were then updated to accommodate risks that could not be coded against v1, producing an updated taxonomy (v2). This coding and updating cycle was repeated iteratively (v3, etc.) until all relevant risks could be coded using the final taxonomy. See supplemental information for detailed documentation of taxonomy iterations.

Seven authors were involved in data synthesis. We used a “best-fit” framework synthesis approach to develop two AI risk taxonomies. Best-fit framework synthesis is a method for rapidly, clearly, and practically understanding the relationships and structures between concepts in a topic area.^22^^,^^23^ It combines the strengths of framework synthesis,^51^ which is a “top-down” positivist method whereby concepts are coded against a pre-existing structure, and thematic synthesis,^52^ which is a “bottom-up” interpretative method whereby concepts are iteratively analyzed to identify patterns and structure. We describe the process in Figure 3.

To conduct a best-fit framework synthesis, we identified published frameworks in an area (through our systematic search and screening), selected the “best” existing framework for our purpose, then used that existing framework to code the concepts (i.e., all the risks extracted into the living AI Risk Database). Some risks could not be coded against the existing framework. We then conducted a secondary thematic analysis to identify new themes in those risks and determined which changes needed to be made to the framework to accommodate those themes. This involved updating the existing categories, creating new categories, or changing the structure of the framework. This process was repeated until achievement of a final version of the framework that could most effectively code all relevant risks.

By starting with an existing framework, the synthesis can achieve a coherent framework more quickly than inductively or thematically analyzing all the individual concepts (risks) across all included papers. The trade-off is that the existing framework creates a particular “lens” for understanding and categorizing the individual concepts, which may lead to a disconnect between the synthesized findings and the theoretical or epistemological perspectives in the original and highly varied papers. To mitigate this, we attempted to code the risks based on the exact wording the authors had presented rather than our interpretation of what they may have intended to communicate (Corbin and Strauss,^49^ cf. Charmaz^50^).

### Why we developed two taxonomies of AI risk

The goal of our best-fit framework synthesis was to create a common frame of reference for understanding and addressing the risks from AI. We found that authors implicitly or explicitly used different lenses^53^^,^^54^ (and, e.g., Head^55^) to create their frameworks. These lenses reveal and obscure different aspects of the AI risk landscape.

Through our systematic search, we identified two types of frameworks, which we refer to here as causal and domain frameworks. “Causal frameworks” focused on antecedents, capturing broad factors that specify how, when, or why an AI risk might emerge (e.g., Critch and Russell^48^ and Kilian et al.^56^) rather than discuss categories of specific hazards and harms. In contrast, “domain frameworks” focused on outcomes, i.e., specific hazards and harms (Weidinger^57^ and, e.g., Solaiman et al.^58^), but did not explore their causes.

The differences here made it challenging to create a single framework. Often, specific domain risks did not fit into the categories within a causal framework, and the broad categories in those frameworks were insufficiently specified to be useful, in isolation, for creating shared understanding. Similarly, broad causal categorizations of how, when, or why an AI risk emerges did not fit with domain frameworks outlining narrow and specific sets of risk.

We therefore resolved that the ideal common frame of reference required two intersecting taxonomies: one to precisely decompose or define an AI risk based on the antecedent conditions under which it occurred (a “causal taxonomy”) and one that classified commonly discussed hazards and harms associated with AI into understandable and distinct domains (a “domain taxonomy”).

In the following sections, we describe the process of developing these two taxonomies using a best-fit framework synthesis approach.

### Development of Causal Taxonomy of AI risks

#### Best-fit taxonomy: Yampolskiy, 2016

We chose Yampolskiy’s “Taxonomy of pathways to dangerous AI” (2016)^29^ as our initial best-fit framework for developing a causal taxonomy for AI risk. We selected Yampolskiy’s taxonomy as it was highly cited (116 citations, fifth most highly cited from the set of identified papers), simple, and comprehensive while providing sufficient definitions for each category.

Yampolskiy’s taxonomy systematically classifies the ways in which an AI system might become dangerous based on two main factors:

(1) Timing—whether the AI became dangerous at the pre-deployment or post-deployment stage, and
(2) Cause—whether the danger arose from external causes (on purpose, by mistake, environment) or internal causes originating from the AI system itself (independently).

Yampolskiy proposes that this taxonomy covers scenarios ranging from AI being purposely designed to be dangerous, to becoming dangerous by accident during development or after deployment, to turning dangerous due to environmental factors outside its control, or evolving to become dangerous through recursive self-improvement. Each “pathway” represents a set of causal conditions that lead to AI causing harm; for example, a person using a large language model (LLM) to generate fake news for political gain would be classified under Path B (“Timing: post-deployment; External cause: on purpose”).

#### Coding and iteration process

We started by using Yampolskiy’s taxonomy to categorize a sample of risks from our database. We then identified themes in the AI Risk Database that did not fit into Yampolskiy’s taxonomy. We updated the taxonomy categories, criteria, and descriptions, then coded a further sample of risks. This process was repeated over three iterations until the taxonomy categories, criteria, and descriptions were stable. We describe this iteration process in detail in Note S3.

#### Final taxonomy

The final version of the taxonomy, which we named the Causal Taxonomy of AI risks, included three categories of causal factors that specify how, why, or when an AI risk might emerge. The first category, Entity, classified the entity (e.g., AI system or human) that was presented as causing the risk to occur due to a decision or action taken by that entity. The second category, Intent, classified whether the risk was presented as an expected outcome or unexpected outcome of an entity pursuing a goal. The third category, Timing, classified the stage in the AI life cycle that the risk is presented as occurring (e.g., pre-deployment or post-deployment). Each of these categories includes a third option, Other, which captures risks that are not clearly categorizable within the primary options. Each of the categories is therefore mutually exclusive; each risk is classified under only one option within each category. The Causal Taxonomy is presented and described in more detail in the results section.

### Development of Domain Taxonomy of AI risks

#### Best-fit taxonomy: Weidinger et al., 2022

We chose Weidinger et al.’s “Taxonomy of risks posed by language models” (2022)^26^ as our initial best-fit framework of consequences because it and its related papers^57^^,^^59^ are among the highest cited in our review. Although this taxonomy was focused on language models, its set of categories was one of the most comprehensive, and it has been iterated upon over several publications. It included six areas of risks from language models.

(1) Discrimination, hate speech, and exclusion
(2). Information hazards
(3) Misinformation harms
(4) Malicious uses
(5) Human-computer interaction harms
(6) Environmental and socioeconomic harms

Each area of risk described several subcategories of risk.

#### Coding and iteration process

We applied this taxonomy by coding as many of the included risks as possible using the Weidinger (2022) taxonomy. We operationalized the taxonomy by using the definitions or descriptions for each category from Weidinger.^26^ Because several similar taxonomies were included in the set identified by the systematic literature review,^26^^,^^57^^,^^59^ we considered descriptions and definitions from any of these taxonomies in our initial coding.

We iterated on the taxonomy to accommodate risks that could not be coded against the existing Weidinger (2022) taxonomy.^26^ The most common risks that could not be accommodated were those related to AI system safety, failures, and limitations; AI system security vulnerabilities and attacks; and competitive dynamics or other failures of governance to manage the development and deployment of AI systems. We describe this iteration process in detail in Note S4.

#### Final taxonomy: Domain Taxonomy of AI risks

The final version of the taxonomy, which we named the Domain Taxonomy of AI risks, included seven domains of AI risk and 24 subdomains of hazards and harms associated with AI. The domains were

(1) Discrimination and toxicity,
(2) Privacy and security,
(3) Misinformation,
(4) Malicious actors and misuse,
(5) Human-computer interaction,
(6) Socioeconomic and environmental harm, and
(7) AI system safety, failures, and limitations.

As with Weidinger’s (2022) taxonomy,^26^ these risk domains are not mutually exclusive; many risks span multiple domains or subdomains due to their interconnected nature. For example, a risk related to AI-generated disinformation could be relevant to both the “Misinformation” domain and the “Malicious actors and misuse” domain. The Domain Taxonomy is presented and detailed in the results section.

### Coding

Three authors were involved in coding risks against our taxonomies. Risks were coded by a single reviewer and discussed with the team where relevant. The coding process involved systematically categorizing each extracted risk according to the definitions within the relevant taxonomy. Based on grounded theory recommendations (cf. Charmaz^50^; Corbin and Strauss^49^), we coded risks as they were presented by the authors, aiming to capture the studied phenomena directly rather than impose our own interpretations or infer intent. When coding risks for our Causal Taxonomy, we categorized risks relevant to multiple levels of each causal factor (e.g., both pre-deployment and post-deployment) as “Other.” In our Domain Taxonomy, we categorized risks relevant to multiple domains and subdomains (e.g., AI-generated disinformation) in the single most relevant category.

### Ongoing expert consultation and updates

We maintain the AI Risk Repository as a living resource through several mechanisms. First, we conduct biannual review cycles in which we screen documents proposed through our public submission form, author outreach, and expert consultations. That is, we solicit new or missed frameworks from the authors of existing frameworks, from interactions with other experts, and from a public form on our website. After screening proposed documents against our criteria, we extract and code risks using the procedures described above. We version the repository with each update logged and dated on the website. For example, between May 2024 and March 2025, we received 44 document recommendations and included 22 that met eligibility criteria, demonstrating active maintenance. Between March 2025 and December 2025, we received 78 document recommendations and included nine that met criteria.

We acknowledge that long-term sustainability depends on continued institutional support. The repository is currently maintained by researchers at MIT FutureTech and the University of Queensland. We have designed the repository infrastructure to minimize maintenance burden: the public submission form, standardized extraction templates, and documented coding procedures allow updates to proceed efficiently. Should our capacity for active maintenance change, we commit to clearly indicating on the website whether the repository remains actively updated or has become a static archive.

## Resource availability

### Lead contact

Further information and requests for resources should be directed to and will be fulfilled by lead contact, Michael Noetel (m.noetel@uq.edu.au).

### Materials availability

This study did not generate new unique reagents or materials.

### Data and code availability

All data are publicly available at https://doi.org/10.17605/OSF.IO/CET8G.^25^

## Acknowledgments

This work was funded by Coefficient Giving (formerly known as Open Philanthropy), who had no role in the design, collection, analysis, interpretation, or reporting of the data.

## Author contributions

Conceptualization, P.S., A.K.S., E.A.C.G., M.N., and N.T.; methodology, P.S., A.K.S., E.A.C.G., J.G., and M.N.; investigation and data collection, P.S., A.K.S., E.A.C.G., and J.G.; analysis, P.S., A.K.S., E.A.C.G., and J.G.; writing—original draft, P.S., A.K.S., and M.N.; writing—review and editing, all authors; project administration, A.K.S., M.N., and N.T.; supervision, M.N., N.T., R.U., J.D., S.P., and S.C.

## Declaration of interests

J.D. and S.P. are employees of Harmony Intelligence, a company that conducts evaluations of AI risks. Their potential conflict of interest did not influence study design, risk classifications, or conclusions.

## Declaration of generative AI and AI-assisted technologies in the writing process

Generative AI (Anthropic’s Claude) was used to provide feedback on the writing in this manuscript. After using this tool, the authors reviewed and edited the content as needed and take full responsibility for the content of the published article.

## Supplemental information

Document S1. Tables S1–S9 and Notes S1–S4

Table S10. Characteristics and risk definitions of documents included in the living AI Risk Database

Document S2. Article plus supplemental information
